# Seizure dynamics: a computational model based approach demonstrating variability in seizure mechanisms

**DOI:** 10.1186/1471-2202-15-S1-P152

**Published:** 2014-07-21

**Authors:** Richard S Balson, Dean R Freestone, Mark J Cook, Anthony N Burkitt, David B Grayden

**Affiliations:** 1 NeuroEngineering Laboratory, Dept. of Electrical & Electronic Engineering, University of Melbourne, Parkville, Victoria, 3010, Australia; 2 Centre for Neural Engineering, University of Melbourne, Carlton, Victoria, 3010, Australia; 3 Department of Medicine St. Vincent’s Hospital Melbourne, University of Melbourne, Fitzroy, Victoria, 3065, Australia; 4 The Bionics Institute, East Melbourne, Victoria, 3002, Australia

## 

Epilepsy is a neurological disorder that affects approximately 1% of the world's population. At present, the underlying mechanisms involved in seizure generation and termination are not fully understood. A computational model-based approach to provide further insights into physiological changes occurring in the brain prior to, during, and post seizure is presented. We demonstrate that an unscented Kalman filter [1] can be used to fit physiological parameters of a neural mass model [2] to recorded EEG. This technique elucidates physiological changes in recorded EEG that cannot be determined with standard EEG analysis methods.

Neural mass models consist of interconnected populations of neurons. They describe the interaction between populations by the mean membrane potential generated by synapses and the firing rate produced by populations.

To demonstrate the ability of this framework to elucidate mechanism involved in seizures, we make use of an *in vivo* model of temporal lobe epilepsy in rats. For the *in vivo* model, tetanus toxin is injected into the rat hippocampus, which results in spontaneously occurring seizures. The effect of the tetanus toxin lasts over a period of 6-8 weeks. We insert a twisted pair electrode into the rat hippocampus to record local field potentials (LFPs). The LFPs are used as the observations for the unscented Kalman filter [3]. The neural mass model considered has been shown to be a good phenomenological model of hippocampal EEG; however it cannot capture high frequency dynamics.

We estimated physiological parameters (synaptic gains) that govern dynamics from four seizures in four animals, based upon recorded LFP 150 seconds prior to and post seizure, as well as during the seizure. The estimation results show that, for three of the four animals, the mechanisms involved in their seizures are similar. For the fourth animal, the estimated physiology prior to, during, and post seizure vary drastically. When comparing the physiological dynamics between animals, we found that the estimation results predicted that there are different mechanisms involved in seizure initiation, evolution, and termination in each animal. The common element in all the results, except for a single estimated seizure, was an increase in excitation at seizure initiation and a decrease at seizure termination.

In the *in vivo* model, the similarity between physiological dynamics observed when considering a single animal, demonstrates that there may be a single mechanism involved in the generation and termination of seizures. However, when considering multiple seizures from different animals, the estimation results show clear differences between the mechanisms involved in seizures. Changes in the estimated synaptic gains at seizure transitions differed between ictal events in an individual rat. This is an unexpected result, as the methodology to model epilepsy in the rats was identical. The estimation results show that it is possible for different mechanisms to be involved in seizures. It is possible that this framework could be used to understand why certain patients are refractory to standard epilepsy treatments. It may further be possible to use this framework to also understand why certain patients become unresponsive to treatment over time.
